# Resume data extract and job recruitment Chatbot features for AI-based resume screening & analytics

**DOI:** 10.1016/j.mex.2025.103775

**Published:** 2025-12-17

**Authors:** Kar Weng Chong, Kok Why Ng, Yong Hong Fu

**Affiliations:** Faculty of Computing and Informatics, Multimedia University, Persiaran Multimedia, 63100 Cyberjaya, Selangor, Malaysia

**Keywords:** Whisper-large, RASA, TF-IDF, BERT, Google Gemini, Word Error Rate (WER)

## Abstract

AI technologies are changing the field of manpower recruitment since they make it much more efficient, accurate, and scalable than conventional approaches. The project builds an AI recruitment system that is combined to have two main elements of an integrated AI recruitment system including a Resume Screening AI and a Job Recruitment Chatbot, which have the goal of improving the process in hiring as well as making the experience of the candidates better in the process.

The Resume Screening AI uses a mixed approach incorporating both classical document processing, and capabilities of advanced language models. The unstructured data of raw resumes are mined and normalized into standard forms to allow evaluation and ranking of the candidates in an organized and systematic manner according to the position’s requirements. The Job Recruitment Chatbot entails a programmed chat system of interactive communication during the job recruitment procedure comprising the component of FAQ, conversation-based direction, and voice-to-text dynamic to make the system more accessible to a diverse group of users.

• **Document Processing Pipeline**: Parsed all-format resumes by the means of PyPDF2 and python-docx libraries and programmed the data in a structured manner, via Google Gemini 1.5 Flash API and engineered special prompts to validate the set of JSON-Schema.
• **Intelligent Screening System**: Created automated candidate screening based on (large language model) inference process to compare resume text with job requirements, producing relevance scores and classified evaluations.
• **Interactive Chatbot Development**: Developed natural language processing AI interface with chat capabilities and with speech-to-text and FAQ automation that could be used to answer candidate questions and optimize the recruitment process.

## Graphical abstract

**Figure:**
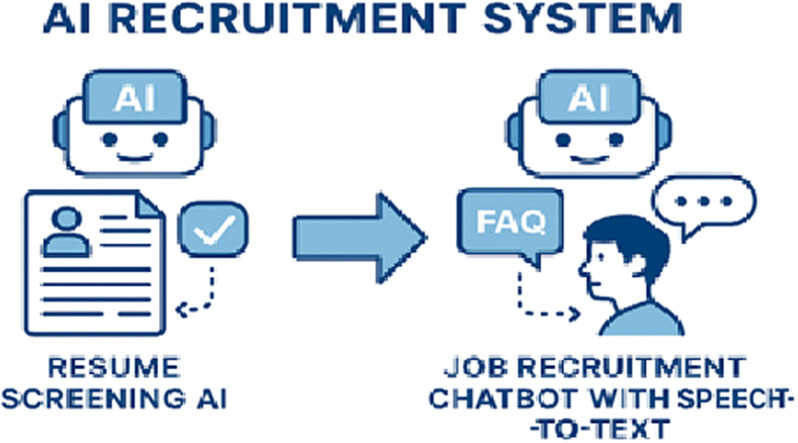

## Methods

Such an integrated system proves to have immense capability in minimizing manual work load and accuracy of a decision and the general efficacy in the recruitment process of an organization at different scales.

## Specifications table

**Table:** 

**Subject area**	Computer Science
**More specific subject area**	Data Science
**Name of your method**	Fine-Tune Whisper-Large, TF-IDF and BERT for Question-and-Answer Validator
**Name and reference of original method**	Fine-Tune Whisper-Large, Fine-Tune BERT
**Resource availability**	https://www.openslr.org/12

## Background

The global digital transformation has seen artificial intelligence (AI) transform many industries like recruitment and analytics. Nevertheless, several issues remain in the way of divine process in the resume screening and recruitment [1].

Moreover, Manual resume Data Extraction is still a time problematic, error prone task. To fill position during the recruitment process there is a big volume of resumes to be filtered, identified and analyzed to candidates’ qualification, skills and experience. This typically delays and is inaccurate, so the ones that are critical information that identify the best candidates may go missing [2].

In addition, interaction modes in job recruitment chatbots are severely limited and hinder inclusivity and user experience. Existing chatbots mostly enable text-based interaction, and candidates who are physically impaired, or have limited language proficiency, may be disadvantaged. Adding a speech to text (STT) function can greatly boost accessibility and comfort which can make a much wider field of candidates using it without a hitch [3].

Accuracy of STT technology poses another challenge. Transcription errors during interaction with a chatbot occur when incoming messages have variations in accents, dialects, speech speeds and background noise, reducing reliability of the chatbot. Consider that it's particularly important that these aren't inaccurate in recruitment cases where the precise answers of candidates are essential so they can judge fairly and effectively [4,5].

Addressing these challenges would enable the efficiency, accessibility, and accuracy of AI based recruitment solutions, making the way organizations screen candidates and make hires [6].

## Method details

### System architecture overview

The AI-powered hiring system consists of four interconnected modules: (1) Resume Extraction (Gemini 1.5 Flash) [7], (2) Speech-to-Text (Whisper-Large) [[8], [9], [10]], (3) Q&A Validation (TF-IDF and BERT) [11,12], and (4) Chat Interface (RASA) [[13], [14], [15], [16]]. Each module functions independently while supporting the overall process.

### Resume data extraction module

• **Text Extraction Process**

The resume pipeline starts with ingesting resumes (PDF, DOC, DOCX) and extracting raw text using OCR and parsing libraries, handling both structured and unstructured formats [17].

• **Gemini 1.5 Flash Integration**

After text extraction, a standardized prompt is generated for the Gemini 1.5 Flash model to extract key resume details (e.g., personal info, education, experience) in JSON format for further processing [7].

• **Data Validation and Error Handling**

JSON files are parsed and validated for format and completeness. Errors triggers retry routines or flag resumes for human review, while valid data is stored in the system database with appropriate metadata [[17], [18], [19]].

### Speech-to-Text processing module

• **Dataset Preparation and Audio Input Processing**

Whisper-Large was fine-tuned on LibriSpeech train-clean-100 and dev-clean sets, split 90:10 for ∼10,000 training and 1000 test samples. Audio was resampled to 16 kHz, with average durations of 12.9 s (train) and 6.6 s (test). Data was imported using parse_librispeech(), ensuring consistency and temporal variation for effective training [[21], [22], [23]].

• **Whisper-Large Model Architecture and Configuration**

**Fig. 1:**
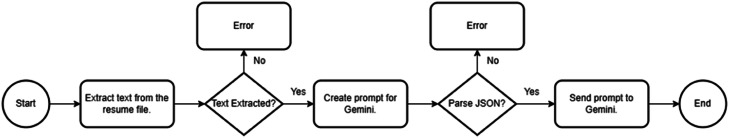
Flowchart of Gemini 1.5 flash model.

The system uses OpenAI’s Whisper-Large model with 24 encoder and decoder layers, fine-tuned for speech-to-text. It includes WhisperFeatureExtractor, WhisperTokenizer, and WhisperProcessor for processing speech and text in a sequence-to-sequence pipeline [8,20] (Fig. 1).

• **Training Configuration and Fine-tuning Process**

**Fig. 2:**
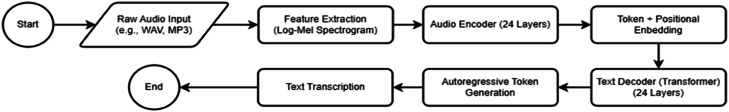
Flowchart fine-tune whisper-large model.

Fine-tuning used Seq2SeqTrainingArguments with batch size 16, learning rate 1e-5, fp16 precision, gradient checkpointing, and 500-step warmup. Training ran for 1000 steps with WER-based evaluation every 500 steps. Padding was handled by a custom collator to preserve attention masks. Losses showed stable training without overfitting [20,24] (Fig. 2, Table 1).

**Table 1:** Training and validation loss for each step.

Step	Training Loss	Validation Loss
500	0.008600	0.112518
1000	0.006400	0.111875

### Question-Answer validation system

• **Dataset Development and Characteristics**

A custom 10,000-sample dataset was created for Q&A validation, covering 10 interview questions with ∼1000 diverse responses each. Each entry includes a question, answer, and binary validity label, with a balanced split of 5000 valid and 5000 invalid samples.

• **Data Preprocessing and Preparation**

Data preprocessing included cleaning, binary label normalization, and stratified 80:20 train-test splitting. TF-IDF used [SEP]-joined Q&A pairs, while BERT used bert-base-uncased tokenizer with 128-token truncation, padding, and attention masking.

• **Dual-Model Implementation and Training**

**TF-IDF Baseline Model**: The baseline used TF-IDF with unigrams/bigrams, stopword removal, and min document frequency of 2. A logistic regression classifier with balanced class weights (liblinear solver) was trained for interpretable comparison [25,27,28].

**BERT Fine-Tuning Architecture**: The BERT model used bert-base-uncased with fp16 mixed precision, batch size 8, and a 2e-5 learning rate. Training was memory-efficient and evaluated per epoch, with the best model selected based on F1-score [11,26,29].

• **Training Results and Performance Monitoring**

**Fig. 3:**
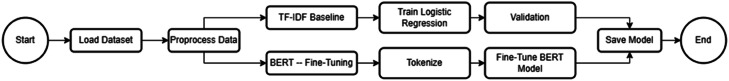
Flowchart of TF-IDF and BERT for Q&A validator.

BERT fine-tuning showed steady improvement over 4000 steps, with training loss dropping from 0.1635 to 0.0031 and evaluation loss from 0.0973 to 0.0443. The final model was selected based on optimal F1-score, indicating effective learning without overfitting (Fig. 3).

### RASA conversational interface

• **Multi-Modal Input Processing**

The RASA chatbot supports both text and speech inputs. Speech is transcribed by Whisper before being processed by the NLU pipeline [14].

• **Natural Language Understanding (NLU)**

The NLU module uses trained models to classify intents and extract entities from recruitment-specific conversations, identifying queries on jobs, interviews, and personal details [30,31].

• **Dialogue Management**

RASA Core manages dialogue flow and context, following predefined paths while adapting to unexpected inputs and follow-up questions [32].

• **Backend Integration and Data Retrieval**

The chatbot connects to the main database to access candidate and job data, enabling real-time, consistent, and dynamic responses across modules [30].

• **Response Generation and Validation**

Before responding, the system validates answers using trained models. Valid responses are delivered via the chatbot, while invalid ones prompt clarifications or escalate to human recruiters (Table 2).

**Table 2:** Training Loss and Eval loss for fine-tuning BERT model.

Step	Training Loss	Eval Loss
1000	0.1635	0.0973
2000	0.0372	0.0752
3000	0.0168	0.0551
4000	0.0031	0.0443

### System integration and workflow

**Fig. 4:**
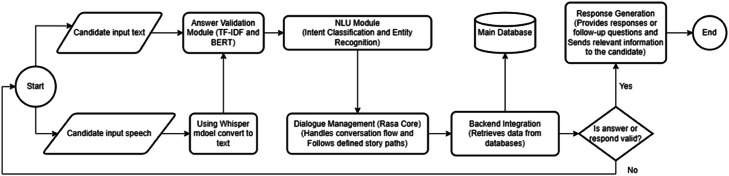
Flowchart of RASA Chatbot.

The four modules work together: resume extraction populates the database, speech-to-text enables voice input, validation ensures response quality, and the RASA chatbot handles interaction. Error handling at each stage ensures system reliability and smooth user experience (Fig. 4).

## Method validation

### Speech recognition model performance

Speech recognition performance was evaluated using three Whisper variants on the LibriSpeech/dev-clean dataset, focusing on Word Error Rate (WER) and average transcription time to assess accuracy and efficiency.

• **Model Comparison Results**

The fine-tuned Whisper-Large model achieved the best WER at 6.03 %, outperforming Whisper-Large-v2 (6.48 %) and Whisper-Small (7.02 %). This 0.45 % improvement highlights the effectiveness of domain-specific fine-tuning.

• **Computational Efficiency Analysis**

**Fig. 5:**
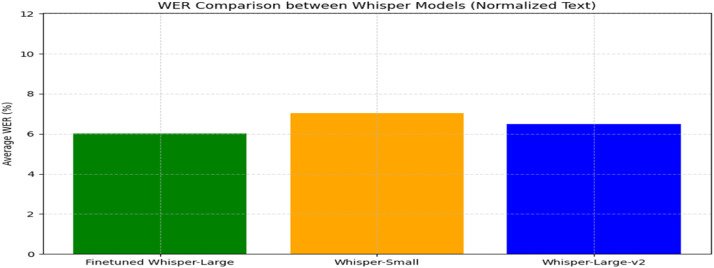
Word error rate comparison between whisper model bar chart.

Transcription time analysis showed a trade-off between speed and model size. Whisper-Small was fastest (0.41s/sample), while fine-tuned Whisper-Large was slower (1.61s/sample). Whisper-Large-v2 balanced both (1.43s/sample), highlighting the need to weigh accuracy against processing speed for real-time use (Fig. 5).

### Question-answer validation system performance

**Fig. 6:**
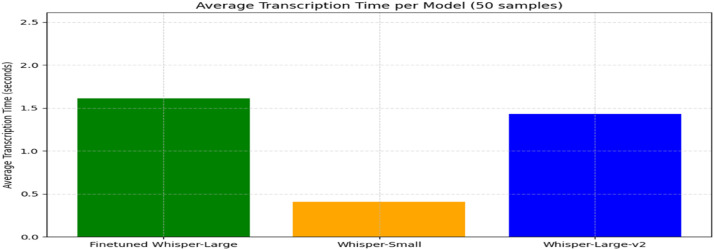
Average transcription time per model bar chart.

To validate the accuracy of automated Q&A system, this project implemented and compared two distinct approaches: TF-IDF with Logistic Regression and Fine-tuned BERT, evaluated on a binary classification task (valid/invalid responses) (Fig. 6).

• **Classification Performance Metrics**

Both models performed strongly across all metrics. TF-IDF + Logistic Regression achieved 95.35 % accuracy, while fine-tuned BERT reached 99.15 %. Matching precision, recall, and F1-scores indicate balanced, unbiased classification performance.

• **Confusion Matrix Analysis**

**Fig. 7:**
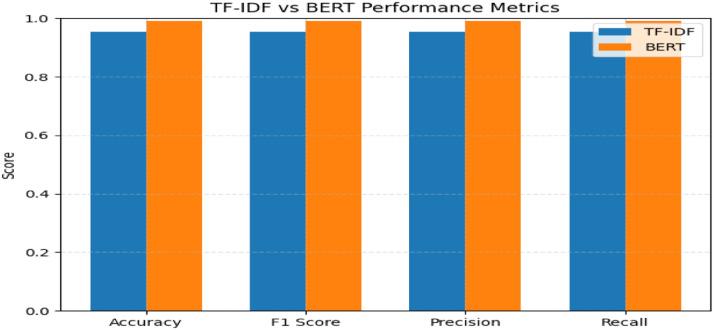
TF-IDF vs BERT performance metrics bar chart.

Confusion matrix analysis confirmed both models’ reliability. TF-IDF achieved strong results with 944 invalid and 963 valid correct classifications, while BERT outperformed with only 8 false positives and 9 false negatives, indicating near-perfect discrimination on 2000 samples (Fig. 7).

• **Model Selection Rationale**

**Fig. 8:**
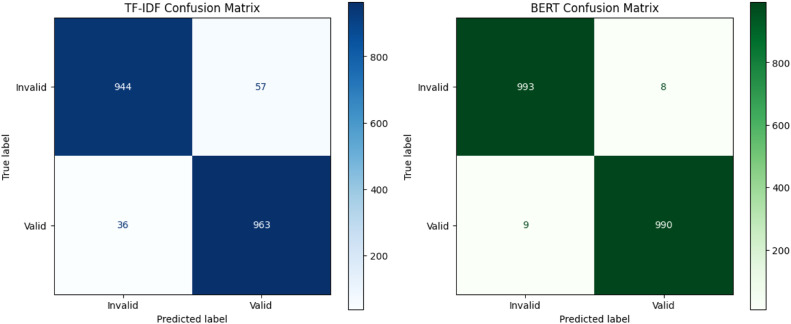
Confusion matrix for TF-IDF and BERT model heatmap.

Between the two models, the fine-tuned BERT achieved higher accuracy (99.15 % vs. 95.35 %) and more balanced error distribution, making it the preferred option for deployment. Its 3.8 % accuracy gain justifies the added computational cost through improved reliability in validation tasks (Fig. 8).

### Validation framework reliability

The hybrid model registered improved performance with 93.97 % accuracy in speech recognition and 99.15 % accuracy in Q&A validation. These results validate the efficiency and operational applicability of the introduced methodology in computerized interview analysis under accuracy constraints within computationally affordable capabilities (Table 3, Table 4).

**Table 3:** Summary table for whisper-large model result.

Model	Dataset	Word Error Rate (WER)	Avg Transcription Time
Fine-Tune Whisper-Large	LibriSpeech/dev-clean	6.03 %	1.61 s
Whisper-Large-v2	LibriSpeech/dev-clean	6.48 %	0.41 s
Whisper-Small	LibriSpeech/dev-clean	7.02 %	1.43 s

**Table 4:** Summary table for Q&A validator model.

Metric	TF-IDF + Logistic Regression	Fine-Tuned BERT
Accuracy	0.9535	0.9915
F1 Score	0.9535	0.9915
Precision	0.9537	0.9915
Recall	0.9535	0.9915

### Limitations

There were system development bottlenecks. Whisper-Large training was a computationally demanding exercise, something even laptops with mediocre capability could not handle—leading to constant crashes, long training time, and space limitation. Monitoring and optimization were needed on an ongoing basis here. Creating an evenly weighted, real-world Q&A set of 10,000 samples took time, had to be populated manually to be linguistically dense and consistent. Finetuning Whisper also involved mass-scale hyperparameter tuning via trial and error to reduce Word Error Rate (WER). Integration of the custom answer validation model into Rasa architecture was also technologically demanding, particularly real-time inference synchronization with dialogue flow, latency control, and consistent validation for multi-turn conversation.

## Related research article

None

## For a published article

None

## Ethics statements

The report does not involve human participants or data from social media platforms.

## CRediT author statement

Chong Kar Weng: Research, Conceptualization, Methodology, Implementation, Writing – original draft, Writing – review & editing

Dr. Ng Kok Why: Writing – review & editing, Supervision

Fu Yong Hong: Writing – review & editing

## Supplementary material and/or additional information [OPTIONAL]

None

## Declaration of competing interest

The authors declare that they have no known competing financial interests or personal relationships that could have appeared to influence the work reported in this paper.

## Data Availability

I already have put in the link of one of the dataset.
